# High lifetime inbreeding depression counteracts the reproductive assurance benefit of selfing in a mass-flowering shrub

**DOI:** 10.1186/s12862-014-0243-7

**Published:** 2014-11-30

**Authors:** Chloé EL Delmas, Pierre-Olivier Cheptou, Nathalie Escaravage, André Pornon

**Affiliations:** Laboratoire Evolution and Diversité Biologique EDB, University Toulouse III Paul Sabatier, F-31062 Toulouse, France; CNRS, EDB, UMR 5174, F-31062 Toulouse, France; UMR 5175 CEFE – Centre d’Ecologie Fonctionnelle et Evolutive (CNRS), 1919 Route de Mende, F-34293 Montpellier Cedex 05, France; Current address: INRA, ISVV, UMR1065 Santé et Agroécologie du Vignoble, F-33883 Villenave d’Ornon, France

**Keywords:** Inbreeding depression, Mixed mating, Perennial species, Self-compatibility, Selfing rate, Reproductive assurance, *Rhododendron ferrugineum*

## Abstract

**Background:**

Decreases in mate and/or pollinator availability would be expected to affect the selective pressure on plant mating systems. An increase in self-fertilization may evolve to compensate for the negative effects of pollination failure. However, the benefit of selfing in variable pollination environments depends on the relative fitnesses of selfed and outcrossed progeny. We investigated the potential for selfing to provide reproductive assurance over the lifetime of a long-lived perennial species and its variation between plant patches of various sizes. Patch size is likely to affect mate and pollinator availabilities, thereby affecting pollination success and the rate of selfing. We estimated fruit and seed set, reproductive assurance, self-compatibility, the multilocus patch selfing rate and lifetime inbreeding depression in natural patches of *Rhododendron ferrugineum* (Ericaceae), a mass-flowering species characterized by considerable patch size variation (as estimated by the total number of inflorescences).

**Results:**

Open seed set declined linearly with increasing patch size, whereas pollinator-mediated seed set (emasculated flowers) was not significantly affected. Progeny array analysis indicated that the selfing rate declined with increasing patch size, consistent with greater reproductive assurance in small sparse patches than in large, dense patches. However, fruit set and adult fitness decreased with decreasing patch size, with an estimated mean lifetime inbreeding depression of 0.9 (obtained by comparing F values in adults and progenies).

**Conclusions:**

Lifetime inbreeding depression strongly counteracts the advantage of reproductive assurance due to selfing in this long-lived species. The poor fitness of selfed offspring should counteract any evolution towards selfing, despite its potential to alleviate the negative consequences of pollen limitation. This study highlights the need to estimate lifetime inbreeding depression, together with mating system and pollination parameters, if we are to understand the actual benefit of selfing and avoid the overestimation of reproductive assurance.

**Electronic supplementary material:**

The online version of this article (doi:10.1186/s12862-014-0243-7) contains supplementary material, which is available to authorized users.

## Background

Plant-pollinator interactions are increasingly affected by human disturbances [1,2], probably resulting in strong selective pressure on plant mating systems [3,4]. Pollination failure may promote the evolution of selfing, which can provide reproductive assurance by increasing seed production when pollinators and/or mates are scarce [5,6]. Reproductive assurance is one of the most longstanding and widely accepted explanations for the evolution of selfing and the maintenance of mixed mating systems [7–11]. If both pollinators and mates are limiting, reproductive assurance can occur through autonomous selfing [9,10]. By contrast, if only mates are limiting, reproductive assurance can also result from pollinator-mediated selfing, such as facilitated selfing [12]. However, inbreeding depression (ID) reduces the fitness of selfed progenies [13,14] and counteracts the advantages of selfing (the 50% intrinsic advantage in terms of pollen transmission [15] and reproductive assurance [5]. The reproductive assurance benefits of selfing therefore depend on the strength of ID. Poor fitness of the selfed progeny may compromise the survival of plant populations with few opportunities to outcross [16]. In conditions of pollen limitation, selfing would be selected for ID values below (1 - *e*) / 2, where *e* is the fraction of ovules remaining unfertilized [17,18].

ID is typically caused either by the expression of lethal deleterious recessive alleles formerly masked by the heterozygous state or by the loss of heterozygote advantage [19,20]. It may evolve with an increase in selfing rate, when deleterious recessive alleles are purged by selection, although this trend is not general in plant species [21,22]. Experimental studies have shown that the expression of ID depends on the environment [23–27]. However, the variability of ID in natural environments and, more specifically, along population size gradients, remains unclear, despite its acknowledged importance [28]. Furthermore, ID estimates are based principally on experimental studies in herbaceous annual species, and the fitness of selfed progenies in long-lived perennials has been little considered [29] but see [30,31]. Perennial species experience multiplicative effects of ID over many years [29] and are exposed to many biotic and abiotic changes.

Given that selfing may be a by-product of outcrossing or provide additional benefits in terms of reproductive assurance in cases of pollination failure, strong ID may be associated with high or intermediate rates of selfing ([32] and references therein). Selfing provides reproductive assurance only if seed production is pollen-limited, if selfing increases seed set and if ID does not completely counteract the benefits of selfing over the lifetime of the adult plant [8,10,32,33]. However, few studies have empirically estimated the actual long-term benefits of selfing by investigating whether the negative impact of ID counteracts the positive effects of the greater seed set due to reproductive assurance in natural populations of contrasting sizes. Given the scarcity of studies simultaneously estimating reproductive assurance, selfing rate and lifetime ID along natural population size gradients, predicting the potential of selfing to provide actual reproductive assurance in various ecological contexts remains a major challenge in evolutionary biology.

We focused on a patchily distributed mass-flowering shrub, *Rhododendron ferrugineum* (Ericaceae). We showed in a previous study that insect visitation rates and floral availability are inversely related, resulting in similar pollinator-mediated pollen transfer in small and large *R. ferrugineum* patches [34]. In this study system, pollen transfer is probably constrained by pollinator limitation in large patches and by mate limitation in small patches, decreasing seed production by 34% on average [35]. Here, we investigated the lifetime reproductive assurance benefit of selfing by estimating reproductive success, reproductive assurance, selfing rate and lifetime ID along a continuum of patch floral display (i.e. the estimated total number of inflorescences in 28 *R. ferrugineum* heathland patches), used as a proxy for patch size. We predicted reproductive assurance and selfing rate to be higher in small sparse patches, in which pollen transfer has been shown to be limited principally by the availability of conspecific plants [34,35]. Lifetime ID (from seed production to maturity) would be expected to be strong in long-lived perennial species and, if associated with early-acting ID, it may counteract the reproductive assurance benefit of selfing in small plant patches. We also characterized the *R. ferrugineum* mating system by estimating fruit set (indicative of early-acting ID) and self-compatibility. We estimated the magnitude of ID in natural populations by using a marker-based approach comparing (i) inbreeding in adult plants (*F* index), including any episodes of mortality in natural populations over the long lifetime of individuals and (ii) multilocus selfing rate (*s*_m_) in progeny arrays, reflecting selfing before selection in natural populations [36,37]. We also estimated the following, along the patch size gradient: (i) pollination parameters, including reproductive assurance, by comparing seed production by emasculated and intact flowers, and self-compatibility, by comparing seed production by hand-selfed and hand-outcrossed flowers and open fruit set; (ii) selfing rate, estimated by progeny array analysis and (iii) ID in natural populations, as assessed with neutral genetic markers.

## Results

### Reproductive success and pollination parameters along a natural patch size gradient

The mean fruit set per patch was 94.5% (range: 80.6 to 100%, Additional file 1: Table S1) and increased significantly with increasing *R. ferrugineum* floral display per patch (*N* =28 patches; *y* =0.05*x* +1.12; estimate =0.05; SEM =0.02; *R*^*2*^ = 0.20. *P* =0.018).

**Figure 1:**
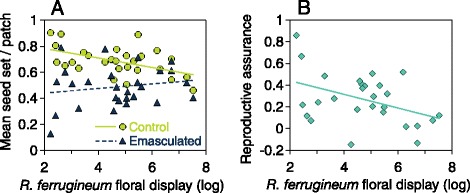
**Effects of** ***R. ferrugineum*** **patch floral display on (A) mean seed set per patch for control flower** ***s (IN*** **treatment,** ***N =28 patc*** **hes;** ***y*** **= −0.037** ***x*** **+** **0.** **857; green circles; solid line) and emasculated flowers (** ***EN*** **treatment,** ***N*** **=27 patches;** ***y*** **=0.017** ***x*** **+0.406; blue triangles; dashed line) and (B) mean reproductive assurance per patch (** ***N*** **=27 patches; RA =1 - emasculated/control;** ***y*** **= −0.035** ***x*** ^**2**^ **+ 0.304x - 0.348).** See Table 1 for methods and statistical comparisons of treatments and Additional file 1: Table S1 for seed sets and pollination parameters per patch.

Mean open seed set (*IN*, Table 1 and Additional file 1: Table S1) decreased significantly with increasing floral display (*N* =28 patches; estimate: − 0.053; SE =0.023; *R*^*2*^ = 0.17; *P* =0.03; Figure 1A) whereas mean pollinator-mediated seed set (*EN*, Table 1 and Additional file 1: Table S1) was not significantly related to patch floral display (*N* =27 patches; estimate: − 0.03; SE =0.03; *R*^*2*^ = 0.05. *P* =0.27; Figure 1A). Open seed set from intact flowers was significantly greater than that of emasculated flowers, consistent with significant reproductive assurance (overall model including the four pollination treatments: F_3,279_ = 51.15; *P* <0.0001; Table 1). Reproductive assurance increased seed production by 27%, on average (Table 1 and Additional file 1: Table S1), and declined significantly with increasing patch floral display (*N* =27 patches; estimate: − 0.06; SE =0.03; *R*^*2*^ = 0.18; *P* =0.029; Figure 1B). Reproductive assurance index could not be calculated for one of the 28 patches, for which *EN* seed set could not be determined.

**Table 1:** **Experimental manipulation for estimating pollination parameters: reproductive assurance (RA) and self-compatibility (SC) estimated with seed sets from four different pollination treatments (** ***IN*** **,** ***EN*** **,** ***IX*** **and** ***ISB*** **)**

**Abbreviation and calculation**	**Definitions**	**Patch mean seed sets/indices**	**Range**	**Tukey-Kramer test**
***Reproductive assurance (RA)***				
***IN***	Seed set from intact inflorescences allowed to undergo natural pollination	0.68	0.46 - 0.9	*IN vs. EN* t =7.86; *P* <0.0001
*N* =28 patches				
***EN***	Seed set from emasculated inflorescences allowed to undergo natural pollination	0.49	0.13 - 0.79	
*N* =27 patches				
**RA =1 – (** ***ENm*** **/** ***INm*** **)**	Proportion of seed production attributable to autogamous self-pollination (including autonomous and facilitated)	0.27	−0.15 - 0.86	
*N* =27 patches				
***Self-compatibility (SC)***				
***IX***	Seed set from intact inflorescences outcrossed by hand	0.71	0.40 - 1	*IX vs. ISB* t = − 9.04; *P* <0.001
*N* =28 patches				
***ISB***	Seed set from bagged intact inflorescences self-pollinated by hand	0.5	0.31 - 0.80	
*N* =28 patches				
**SC =** ***ISBm*** **/** ***IX*** **m**	Early-acting ID or partial self-incompatibility effect on seed set in selfed flowers	0.71	0.53 - 1	
*N* =28 patches				

Patch means, ranges and Tukey-Kramer tests are presented to compare *IN vs. EN* (reproductive assurance) and *IX vs. ISB* (self-compatibility). See the Method section for model description and Additional file 1: Table S1 for raw data per patch.

The mean seed set for hand-outcrossed flowers was significantly higher than that for hand self-pollinated bagged flowers (*IX* and *ISB*, respectively; Table 1 and Additional file 1: Table S1), indicating partial self-compatibility. Neither of these seed set values was significantly related to patch floral display (*N* =28 patches; *IX*: estimate = − 0.03; SE =0.03; *R*^*2*^ = 0.04; *P* =0.31; *ISB*: estimate = − 0.01; SE =0.02. *R*^*2*^ = 0.003; *P* =0.77). Self-compatibility (Table 1 and Additional file 1: Table S1) was not significantly related to floral display (*N* =28 patches; estimate =0.01; SE =0.02; *R*^*2*^ = 0.01; *P* =0.69).

### Patch selfing rate estimates

Significant amounts of selfing were observed in all patches (Table 2). Selfing rates not significantly different from 0 were found in two patches (patches 8 and 13), as indicated by the 95% confidence limits of the bootstrap distribution. Overall, *R. ferrugineum* has a mixed mating system, with a mean multilocus selfing rate per patch (estimated as 1 - *t*_*m*_) of *s*_*m*_ =0.49 (range: 0.21 ± 0.069 to 0.84 ± 0.294; Table 2). Selfing rate decreased significantly with increasing *R. ferrugineum* floral display (estimate = − 0.05; SE =0.023; *R*^*2*^ = 0.156; *P* =0.037).

**Table 2:** **Mating system estimates (multilocus patch selfing rate**, ***s***_**m**_ **), Wright’s fixation index (** ***F***_**IS**_ **) and lifetime inbreeding depression (ID) for 28** ***Rhododendron ferrugineum*** **patches from the Pyrenees (France) surveyed in 2009**

	**Mating system estimates** ***s***_**m**_ **(1 -** ***t***_**m**_ **)**			**Wright’s fixation index** **(** ***F***_**IS**_ **)**			**Lifetime ID**	
	***N***_**f**_	**Mean**	**SE**	***N***_**i**_	**Mean**	**95% CI 1000 bootstraps**	**Mean**	**95% CI 1000 bootstraps**
Patch 1	2	0.54	0.17	13	0.10	−0.03/0.16	0.81	0.44/1.18 c
Patch 2	3	0.73	0.16	3	-	-	-	-
Patch 3	3	0.52	0.22	4	−0.15	−0.58/0.10	1.24	−4.81/7.29
Patch 4	3	0.84	0.07	17	0.05	−0.09/0.13	0.98	−0.13/2.09 c
Patch 5	3	0.54	0.12	3	-	-	-	-
Patch 6	2	0.66	0.30	2	-	-	-	-
Patch 7	4	0.70	0.05	16	0.05	−0.11/0.149	0.95	0.46/1.45 c
Patch 8	3	0.21 a	0.29	3	-	-	-	-
Patch 9	5	0.38	0.13	35	−0.01	−0.12/0.08	1.03	0.75/1.31
Patch 10	4	0.34	0.24	20	0.07	−0.06/0.17	0.70	0.35/1.05
Patch 11	4	0.52	0.22	20	−0.02	−0.15/0.07	1.03	−0.3/2.36
Patch 12	4	0.55	0.09	20	−0.01	−0.17/0.10	1.01	0.07/1.96
Patch 13	4	0.24 a	0.26	20	0.04	−0.08/0.10	0.70	−0.01/1.42
Patch 14	5	0.53	0.10	55	0.05	−0.04/0.11	0.91	0.58/1.25
Patch 15	4	0.24	0.16	20	0.00	−0.13/0.08	0.97	0.09/1.85
Patch 16	4	0.24	0.21	20	0.22 b	0.05/0.33	−0.43	−0.48/0.39 d
Patch 17	3	0.73	0.43	20	0.10 b	−0.05/0.19	0.92	0.18/1.66
Patch 18	4	0.62	0.10	20	0.06	−0.09/0.16	0.92	0.2/1.64 c
Patch 19	4	0.34	0.19	19	0.02	−0.14/0.11	0.94	−0.19/2.07
Patch 20	6	0.65	0.09	20	0.09 b	−0.06/0.18	0.90	0.88/0.92
Patch 21	5	0.69	0.14	19	−0.02	−0.16/0.04	1.03	−0.69/2.73
Patch 22	4	0.74	0.07	19	0.01	−0.12/0.07	1.00	0.37/1.62
Patch 23	4	0.66	0.17	20	0.07	−0.06/0.15	0.92	0.48/1.35
Patch 24	4	0.27	0.13	20	−0.04	−0.17/0.02	1.21	0.01/2.41
Patch 25	4	0.32	0.25	30	0.03	−0.09/0.10	0.89	0.13/1.64
Patch 26	3	0.34	0.14	14	0.06	−0.12/0.15	0.75	−0.03/1.53
Patch 27	4	0.25	0.19	16	−0.03	−0.14/0.01	1.17	0.08/2.26
Patch 28	4	0.27	0.18	14	−0.02	−0.16/0.05	1.09	−0.14/2.32

Sample sizes (*N*_f_: number of families, *N*_i_: number individuals), means and standard errors (SE) or 95% confidence intervals (CI) are presented. Patches are classified from the smallest (Patch 1) to the largest patch floral display (Patch 28). Four patches had a small number of individuals (*N*_i_), resulting in an irregular bootstrap distribution of *F* estimates. The estimates of *F* and ID for these four patches are, therefore, not presented (-). See the Method section for lifetime inbreeding depression calculation.

*Abbreviations:* *t*_m_: multilocus patch outcrossing rates, *s*_m_: multilocus patch selfing rates (*s*m =1 - *t*m); *N*f: number of families; SE: Standard errors; *N*i: number of individuals; CI: confidence interval; ID: inbreeding depression; a: patch selfing rate not significantly different from 0 based on 95% confidence intervals. b: *F* significantly higher than zero (more homozygotes than expected at Hardy–Weinberg equilibrium) based on 95% confidence limits. c: bootstrap distribution of ID values significantly different from 1. d: bootstrap distribution of ID values significantly lower than 0. All other patches had ID values significantly higher than 1.

### Lifetime inbreeding depression

**Figure 2:**
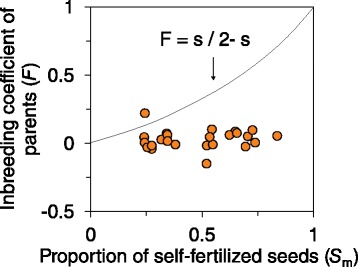
**The relationship between estimated levels of self-fertilization in progeny (** ***s***_***m***_ **=1 −** ***t***_***m***_ **) and the inbreeding coefficient (** ***F*** **) of adult** ***R. ferrugineum*** **individuals in heathland patches.** *N* =24 patches (estimates of *F* for four patches are not presented because bootstrap distributions were irregular due to the small number of individuals per patch; see Table 2). The solid line shows the expected relationship between *s* and *F* in patches at equilibrium (*F*e) with no ID (ID =1 − [fitness of selfed progeny/fitness of outcrossed progeny] =0). In the case of ID, data points are below this solid line.

Estimates of ID reached 0.9 ± 0.03 (SE) on average (Table 2). Based on the distribution of ID bootstrap values, only four patches had values significantly lower than 1 (Wilcoxon tests; Table 2). Values above 1 were due to negative *F* values (i.e. more heterozygotes than expected under the assumption of Hardy–Weinberg equilibrium). In one patch, the rate of selfing for the mother plants exceeded that of the progeny (F =0.22; *s*_*m*_ =0.24), resulting in a negative value for ID (see the methods for the appropriate ID estimate). We found no evidence for a relationship between the strength of ID and patch floral display (*N* =24 patches; estimate =0.007; SE =0.047; *R*^*2*^ = 0.001; *P* =0.89). The mean observed inbreeding coefficient of mature plants (*F*) was 0.04 (95% bootstrap percentile confidence interval based on 1000 bootstraps: −0.15 to 0.21). *F* was not significantly different from zero in 21 of the 24 patches, indicating that the selfed progeny contributed little to the adult population (Table 2). The observed inbreeding coefficients (*F*) of mature plants were almost always lower than expected given the level of self-fertilization (*s*_*m*_) in natural conditions (the expected relationship between adult *F* and *s*_*m*_ is *F* = *s*_*m*_ / (2- *s*_*m*_); Figure 2). Finally, there was no evidence of a relationship between *F* estimates and patch floral display (*N* =24 patches; estimate = − 0.001; SE =0.01; *R*^*2*^ = 0.0002; *P* =0.95).

## Discussion

The level of selfing [38,39] and reproductive assurance [10,40] have been shown to be influenced by the availability of pollinators and/or mates. However, the benefits of selfing in terms of the reproductive assurance provided when pollinators and/or mates are scarce is dependent on selfed-progeny fitness *in natura*. Our results demonstrate that seed production and reproductive assurance increase with decreasing patch size. Furthermore, pollinator-mediated seed set was not affected by patch size. Almost half the progeny (49%) were produced by selfing, on average, and the rate of selfing increased with decreasing patch size. However, the reduced fruit set in small patches and the strong mean lifetime ID of 0.9 strongly counteract the reproductive assurance benefit of selfing.

### Reproductive assurance and selfing rate estimates for patches of different sizes

We found that autogamy significantly increased seed set, as previously shown for other systems [41–44]. We showed in a previous study that flower visitation rates increase [34] and pollen limitation decreases [35] with decreasing patch size, as a result of pollinator monopolization by the mass-flowering plant *R. ferrugineum* in small patches and intraspecific competition for pollinator services in larger patches. Reproductive assurance (mean value of 27%) enhanced *R. ferrugineum* reproductive success in small patches in response to the low availability of conspecific plants with no decrease in pollinator availability. Mate limitation has been shown to be an important driver of plant mating systems [45,46], potentially resulting in the evolution of simultaneous autogamy (autonomous or facilitated) with moderate pollen and seed discounting [3]. The level of self-compatibility was not affected by patch size (despite variation within the study site) and cannot, therefore, be responsible for the variation in reproductive assurance along the patch size gradient. The greater reproductive assurance in small patches is more likely to result from facilitated self-pollination, as suggested in the case of mate limitation in a previous study [3].

The perennial mass-flowering species studied has a mixed mating system (mean *s*_*m*_ =0.49). The selfing rate in *R. ferrugineum* is higher than that in other species from the Ericaceae (mean of 0.2 in both *R. aureum* [47], and *Calluna vulgaris* [48] and it increased with decreasing patch size. Thus, more seeds were produced by selfing in small patches than in larger patches, probably due to the lower availability of mates and/or weaker early and lifetime ID. Selfing rates have been shown to respond to ecological factors and to decrease with increasing plant density [39,49] and to increase with increasing patch floral display [50]. In addition, we found that selfing rates were higher than expected from reproductive assurance alone (mean of 0.49 *versus* 0.27, respectively). This indicates that, in addition to reproductive assurance (taking both autonomous and facilitated selfing into account in the estimation), geitonogamy was an important additional source of self-pollen. Reproductive assurance is particularly costly when associated with ovule usurpation by selfing in situations in which outcrossing could otherwise occur (seed discounting) [51]. Pollinator-mediated pollen transfer decreased seed production by 34% on average in this system (comparing the seed set of emasculated and pollen-supplemented flowers; [34], so we consider the risk of seed discounting to be moderate (see [3] for a hypothesis concerning the consequences of mate limitation).

### Early-acting and lifetime inbreeding depression in a mass-flowering shrub

The reduced fruit set in small patches, the mean self-compatibility index of 0.71 and its variability between individuals (CV of 47%) suggest that ID acted early on fruit set in small patches [52–54] and was probably mediated by a small number of few mutations of major effect. A similar situation has already been reported in the Ericaceae [48,55], resulting in higher mortality between fertilization and seed maturation for selfed zygotes than for outcrossed zygotes.

Lifetime estimates of ID *in natura* were very high (mean: 0.9), not significantly affected by patch size, and well above the threshold value of 0.5 below which selfing is favored due to its transmission advantage [13,56]. This threshold, which was proposed for annuals [6], applies to perennials only for lifetime estimates of ID, such as those obtained here [29]. Our results suggest that there is strong selective pressure against selfed progenies, as already reported for other perennials [8,57,58]. An association of strong ID with high levels of self-incompatibility and a predominance of outcrossing is commonly observed in long-lived species [59–61], due to the accumulation of somatic mutations over the lifetime of the organism ([29,62] but see also [63]. By contrast, in the species studied here, strong lifetime ID and early-acting ID were associated with substantial selfing and mixed mating systems. This theoretical paradox has been reported in several species (reviewed in [32,37,64].

Theoretically, ID should decrease with increasing selfing rates, due to the purging of deleterious recessive alleles [52,65]. In this context, we might have expected the relationship between ID and patch size to depend on selfing rate. However, strong ID may be maintained despite high selfing rates if there are mildly deleterious alleles at multiple loci [21,66,67]. More specifically, selective interference [68,69], preventing the purging process [64], could account for stable mixed mating associated with strong ID in natural populations, even if the selfing rate is high [70]. Selective interference is likely in perennial species, such as *R. ferrugineum,* because it increases the rate of mutation per generation [71]. ID appears to be extremely costly in *R. ferrugineum*, particularly in small patches, in which most of the seeds are produced by selfing.

### Reproductive assurance *versus* inbreeding depression

The actual benefit of selfing, in terms of the reproductive assurance it provides, therefore depends heavily on the fitness of the selfed progeny, particularly in long-lived species. The potential for reproductive assurance is therefore likely to be overestimated if the pollination parameters and the actual mating system are not assessed together with lifetime ID. The causes and consequences of this best-of-both-worlds strategy (i.e. selfing provides reproductive assurance but is associated with ID, leading to sexual reproduction to maintain genetic variability in mating systems) have been little studied [72,73].

Mixed mating systems may be favored by the ecological context [37], as the benefits of reproductive assurance can outweigh the disadvantages of ID if the success of outcrossing is limited by low pollinator and/or mate availability [5,74–79]. Reproductive assurance may, therefore, be essential for species forming small populations in subalpine environments, in which there may sometimes be a shortage of pollinators [10]. However, reproductive assurance may be less advantageous in long-lived perennials, in which a failure to reproduce in some years does not compromise lifetime fitness [80]. We found that reproductive assurance clearly increased seed production in small patches in which mates were scarce, but the gain was almost entirely cancelled out by ID (see also [8]).

By contrast, stochastic environments, such as high-altitude habitats, may select for outcrossing, leading to high levels of genetic variability and favoring population persistence [81]. The commonly reported low abundance and activity of pollinators in such systems may lead to the evolution of stronger interactions with pollinators via large floral displays [4]. The mass-flowering habit of *R. ferrugineum* clearly enhances pollinator attraction [34], but it also illustrates the conflict between self- and cross-fertilization [82,83]. Selfing in trees and shrubs may be an unavoidable consequence of insect visitation [71,84] and may be autonomous or geitonogamous and facilitated (a by-product of attractiveness to pollinators). Visitation rates tend to be higher in small patches [34], probably increasing both facilitated self-pollination and geitonogamy [85,86] and, possibly, pollen [87] or seed discounting [17]. If not opposed by early-acting and lifetime ID, the establishment of seedlings from seeds produced via facilitated selfing or geitonogamy may have an impact on the genetic structure of populations [88]. In *R. ferrugineum*, massive flowering and buzz pollination have positive effects on pollinator attraction and pollen delivery, favoring either outcrossing or selfing (in large and small patches, respectively). However, strong lifetime ID preserves heterozygosity along the patch size gradient in mature populations.

## Conclusions

The abundances of pollinators and plant species are currently decreasing in parallel [2], and the adaptive evolution of plant mating systems towards self-pollination in response to these changes has been predicted [4]. However, our results suggest that, in species like *R. ferrugineum*, ID strongly counteracts the reproductive assurance benefit of selfing. In the case of outcross pollen limitation in the presence of such ID, small and sparse populations may therefore be at risk of extinction [71]. The higher selfing rates found in small patches appeared to be non-adaptive with respect to lifetime ID and to be a by-product of the pollination context, which may reflect recent patch fragmentation. The intriguing paradox of a high selfing rate despite high lifetime ID should be considered in the context of species’ range scale estimates, because both selfing rate and ID may vary with the environment. This study highlights the need to estimate lifetime ID along with the actual mating system and pollination parameters if we are to understand the real benefit of selfing and avoid overestimating the benefits of reproductive assurance.

## Methods

### Study organism

**Figure 3:**
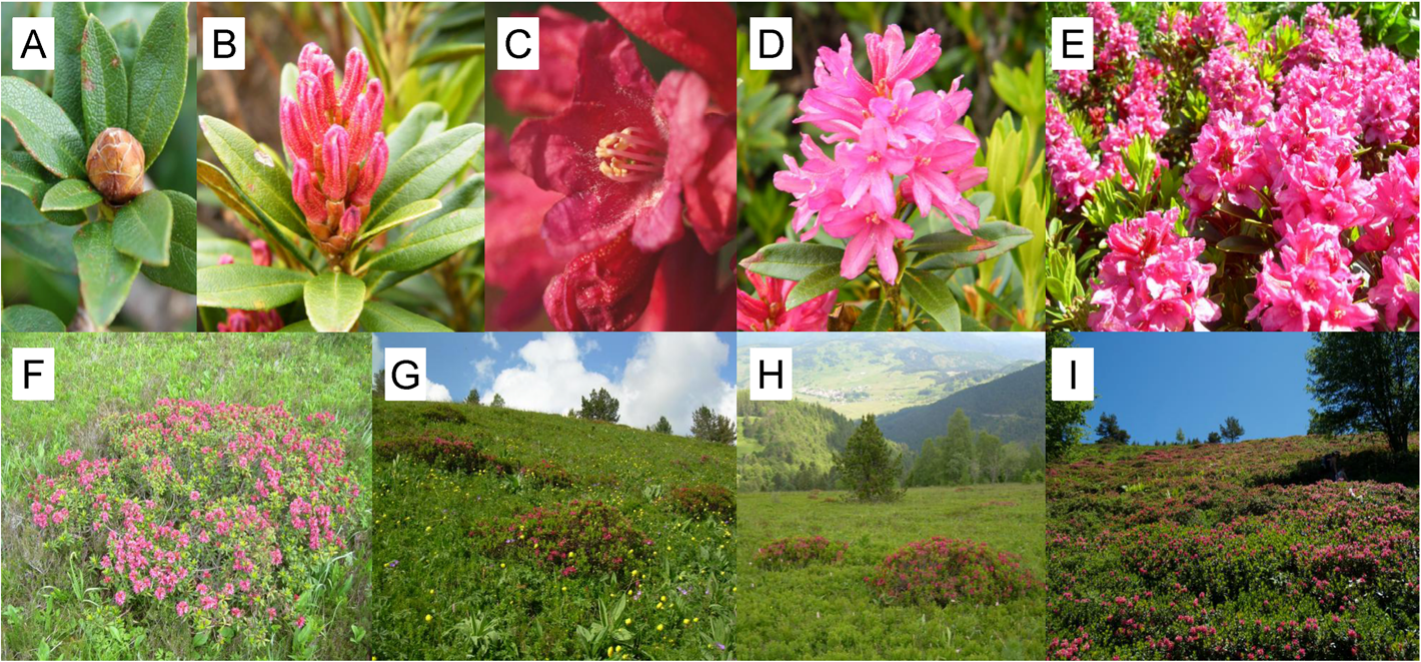
**Flower and patch structure of** ***Rhododendron ferrugineum*** **. (A)** Inflorescence bud. **(B)** Inflorescence before blooming. **(C)** Bright red nectariferous tubular flowers with poricidal anther dehiscence. **(D)** Inflorescence in bloom. **(E)** Massive floral display. **(F)** One individual shrub. **(G)** and **(H)** are small patches. **(I)** is a large closed patch.

*Rhododendron ferrugineum* L. (Ericaceae) dominates and structures heathland communities on nutrient-poor soils in Europe at elevations of between 1400 and 2300 m a.s.l., particularly in the Alps and Pyrenees [89]. Age-old burning and livestock grazing practices have resulted in heathlands being fragmented, resulting in highly variable patch sizes and isolation (Figure 3). *Rhododendron ferrugineum* is an evergreen shrub that is self-compatible, capable of autonomous autogamy and of both sexual and asexual reproduction [90]. Its most efficient pollinators are honey bees and bumblebees [91]. A list of foraging insects observed visiting *R. ferrugineum* at the study site was published in a previous study [34]. This plant has a massive floral display (Figure 3), reaching more than 300 inflorescences of about 11 bright red nectariferous tubular flowers with poricidal anther dehiscence [90]. The flowers present stamen dimorphism, with an inner whorl of five long stamens and an outer whorl of five short stamens [92]. The flowering period of a patch is 20–30 days long (June - July) and the flowers, each lasting about 10 days, are initiated the year before they mature.

### Study site and patch characteristics

The study was conducted on a 3 km^2^ area in the French Central Pyrenees, at an elevation of 1550 m to 1750 m a.s.l., near the village of Camurac (42°46′31″N 01°55′45″E). We used the 28 *R. ferrugineum* heathland patches found at this location because they display a broad gradient of patch size. We considered a heathland patch to be a visually distinct aggregation of *R. ferrugineum* shrubs separated from another patch by meadow. The study site and its patch structure have been described elsewhere [34,35]. We approximated patch size by determining the total *R. ferrugineum* floral display per patch. To describe *R. ferrugineum* floral display in each heathland patch, we integrated inflorescence density, the cover of the species and patch area. More specifically, *R. ferrugineum* patch floral display was estimated through the product of (i) the mean density of inflorescences per m^2^ assessed from a 0.25 × 0.25 m plot placed on 20 randomly chosen individuals per patch and (ii) the area (m^2^) covered by *Rhododendron* within each patch estimated from the total area of the patch (patch perimeter obtained from geographic coordinates recorded every 5 meters) and the proportion of this total area occupied by *R. ferrugineum*. This latter was estimated by summing perimeters of all *Rhododendron* individuals in a 400 m^2^ plot haphazardly placed at the patch core. Within patches, the percentage of *R. ferrugineum* cover ranged from 0.18% to 98% and total patch area ranged from 0.01 to 15.77 ha (1.73 ± 0.38 ha). Overall, the estimation of *R. ferrugineum* patch floral display ranged from 170 to more than 33 × 10^6^ rhododendron inflorescences in the largest patch of 16 ha (mean: 2,529,257).

### Reproductive success and pollination parameters along a natural patch size gradient

We estimated the pollination and reproductive characteristics of *R. ferrugineum*, by selecting109 individuals at random along the natural patch size gradient (four individuals per patch if possible). We chose to maximize the number of patches (our sampling units) to encompass a broad gradient of local pollination environments. In June 2009, we performed four pollination treatments (Table 1), replicated twice on each target individual in each patch (total sample size of 872 inflorescences overall), as previously described [3]. The two inflorescences per treatment were selected at random on each individual. The mean number of inflorescences per individual was estimated at 851.2: we counted all inflorescences per individual. However, when the individual was very large, we quantified the number of inflorescences on a 25×25 cm square and then used the perimeter of the individual to estimate the total inflorescence number per individual (inflorescence density is homogeneous within *R. ferrugineum* individuals because all inflorescences are on the top of the shrub). Five flowers per inflorescence were manipulated, and we gently removed the other flowers, as previously described [34,35,90]. This had no effect on the production of seeds by the remaining flowers [93]. For the *EN* treatment, emasculation was performed by excising the anthers before anthesis, to prevent the deposition of self-pollen: an emasculated flower sets seeds only if it has been visited effectively by pollinators (pollinator-mediated seed set). For the *IX* treatment, flowers were supplemented with outcross pollen every other day. The pollen used was collected from a distant individual (more than 5 m away) from the same patch. For the *ISB* treatment, we bagged inflorescences at the bud stage and hand self-pollination was performed by gently moving the anthers against the stigma within a flower.

We estimated mean fruit set for each target individual by counting the number of filled fruits over the total number of fruits (filled and aborted) from the two *IN* inflorescences per individual.

We estimated mean seed set per treatment per target individual, by harvesting fruits just before dehiscence, to ensure the full development of the seeds. Two fruits per manipulated inflorescence were selected at random and the filled seeds were counted under a stereomicroscope. It is not possible to count aborted seeds in *R. ferrugineum* because they are too small (<1 mm and indistinguishable from small pieces of the dissected fruit). We therefore used a stereomicroscope to determine the number of ovules per ovary in four flower buds per individual preserved in 70% alcohol. Two flower buds were collected from each of two inflorescence buds. On average, each flower produced 481 ± 106 ovules. We calculated the mean seed set per treatment per target individual by dividing the mean number of mature seeds by the mean number of ovules of the same individual.

We estimated the reproductive assurance benefit of selfing per patch (Table 1), as previously described [94]. Positive reproductive assurance values indicate that autogamy provides reproductive assurance, whereas values of zero and negative values indicate an absence of reproductive assurance benefit. A previous experiment in the Alps showed that seed set did not differ significantly between emasculated flowers and unmanipulated flowers [90]. Emasculation therefore probably has no significant effect on pollinator attraction in *R. ferrugineum*. We assessed the level of self-compatibility for each patch (Table 1), by dividing seed set from manually self-pollinated flowers by seed set from cross pollen-supplemented flowers from the same patch [94].

We assessed the importance of reproductive assurance and self-compatibility, by comparing seed set between the four pollination treatments. We used a mixed model, with the individual as a random factor, to account for the non-independence of treatments on the same individual. For comparisons of *EN* and *IN* (reproductive assurance) and of *ISB* and *IX* (self-compatibility), we performed a Tukey test on treatment least squares means, using the approximation described in [95] to adjust for multiple comparisons (“Tukey-Kramer test” performed with PROC GLIMMIX). We carried out linear regression analyses to investigate the relationship between *R. ferrugineum* floral display and reproductive and pollination estimates (fruit and seed sets, reproductive assurance and self-compatibility). The analysis was based on mean values for each patch. *R. ferrugineum* floral display was log-transformed to reduce the impact of very large floral displays (>30 million inflorescences) with respect to that of other patches in the model. Fruit and seed set were arcsine square root-transformed before analysis, to obtain a Gaussian distribution. Statistical analyses were performed with PROC REG in SAS (version 9.2; SAS Institute, Cary, North Carolina, USA).

### Progeny array analysis and selfing rate estimates

In July 2009, we collected young leaves from the 109 target mother plants (Table 2). Leaf material was conserved in silica gel until DNA extraction. In August 2009, we collected mature fruits from these plants for progeny array analyses. We randomly chose several unmanipulated fruits from each individual to obtain open-pollinated seeds (all seeds were pooled). Three of the mother plants were not sampled because the fruits had already dehisced at the time of seed collection. *R. ferrugineum* seeds are 1 to 2 mm long. We germinated an unknown number of seeds from each mother plant on moistened filter paper in Petri dishes in the greenhouse. The greenhouse conditions made it possible to minimize ID at the germination stage [25], thereby reducing the potential bias in outcrossing rate estimates. Ideally, we collected 10 seedlings per family selected at random once the cotyledons had developed. These seedlings were then frozen until DNA extraction (mean of 9.6 seedlings per family), for the estimation of patch selfing rate. Germination rate was high (close to 100%), but was not precisely monitored as this was not the purpose of the study. The total sample size was 106 families, including 1001 progeny for mating system analysis. The protocol for DNA extraction, and for the PCR amplification and genotyping of 12 polymorphic microsatellite loci has been described in detail elsewhere [35,96]. Nine of the 12 loci were developed through pyrosequencing technology [96]: RF6P2, RF14P3, RF38P1, RF41P1, RF46P2, RF47P1, RF56P1, RF74P1 and RF81P1. Two were developed in *Rhododendron metternichii* [97]: RM3D2 and RM2D2, and one in *Rhododendron simsii* [98]: AZA-003. In this study, the mean number of alleles per locus was 8.2 (ranging from 2 to 18; combining samples from outcrossing rate and ID analyses - see below).

Single-locus and multilocus patch outcrossing rates (*t*_*s*_ and *t*_*m*_, respectively) were calculated with the MLTR program [99], with Newton–Raphson iteration, which fits the observed proportions of genotypes descended from a known maternal genotype to the proportions expected under the mixed-mating model [100,101]. We calculated standard errors of multilocus patch outcrossing rates as the SD of 1000 replicate bootstrap estimates, with the progeny array as the unit of resampling. Multilocus patch selfing rates (*s*_*m*_) were calculated as *s*_*m*_ =1 – *t*_*m*_. The program used can estimate allelic frequencies separately for the pollen and ovule pools. As no significant difference was found between pollen and ovule allele frequencies, we constrained the equality of frequencies to increase the statistical power of other estimates [99]. The analyses were performed with patch identity as a group factor and the estimates *t*_*s*_ and *t*_*m*_ were obtained for each patch (*N* =28). Finally, we used a linear regression (PROC REG) analysis to investigate the relationship between *R. ferrugineum* floral display (log) and multilocus selfing rates (*s*_*m*_).

### Lifetime ID

We assessed lifetime ID in natural patches, from seed to reproductive maturity, by comparing the inbreeding coefficients (*F*) of mature plants with the expected *F* of progeny based on selfing rate (*s*_*m*_ =1 – *t*_*m*_). The difference between inbreeding rates for the seedlings and adults provides an indication of the strength of the ID counteracting selfing in each patch [36,37]. We assessed inbreeding coefficients for the adult plants in each patch, by collecting leaf samples from a mean of 18 randomly chosen individuals (all adults were collected if there were fewer than 20 individuals per patch, see Table 2). The total sample size for inbreeding coefficient analysis was 502 adults. DNA extraction and amplification were performed as described above. We screened these 502 adults for clones; only 2.2% of individuals were found to be genetically identical (three patches had two genetically identical individuals and one patch had two clonal colonies consisting of three and two genetically identical individuals, respectively). We retained only one of the genetically identical individuals per clonal colony. We therefore considered all the sampled individuals to be genetically independent. We calculated the inbreeding coefficients (*F*) of mature plants with GENETIX v4.05.2 [102] and the significance of *F* values was determined by applying 1000 randomizations. Four patches consisted in small numbers of individuals, resulting in an irregular bootstrap distribution of *F* estimates. The *F* and ID estimates for these four patches are, therefore, not presented (Table 2).

In the absence of ID, the expected equilibrium value of *F* for mature plants is *F*e = *s*_*m*_*/* (2 − *s*_*m*_). As previously described [37], ID reduces *F* to *F*e = *s*_*m*_ ω_s_*/*(2 − 2*s*_*m*_ + *s*_*m*_ω_s_), where ω is the fitness of selfed offspring relative to outcrossed offspring (i.e., ω_s_ =1 − ID). Ritland’s equilibrium estimator [36] was therefore used to estimate ID as:

$$ \mathrm{ID}=1-2\left[\frac{\left(1-{s}_m\right)F}{s_m\left(1-F\right)}\right] $$

This estimator of ID assumes that populations are at inbreeding equilibrium, selfing is the only form of inbreeding, the marker polymorphisms are neutral and not physically linked to polymorphic loci affecting fitness and there is no identity disequilibrium [36,103,104].

Ritland’s ID estimator is appropriate for positive ID values, but it ranges from -∞ to 0 when the fitness of the selfed offspring exceeds the fitness of the outcrossed offspring (ω_s_ > ω_o_), giving a disproportionate weight to negative ID values. We generated a distribution of ID that was symmetric, regular and suitable for use to sum ID estimates in an unbiased fashion in cases in which the self progeny outperformed the outcross progeny, by using a previously derived alternative estimate of ID [105] that has been used in other studies ([106–110] among others). When ω_s_ > ω_o_ (i.e. Ritland’s equilibrium estimator <0), we used the following ID estimate: ID = (ω_o_ – ω_s_)/ω_s_ instead of the usual ID = (ω_o_ - ω_s_)/ω_o_. We considered the fitness of outcrossed progeny to be 1: ID = (1/ ω_s_) - 1. The estimate of ID used in such cases was therefore:

$$ \mathrm{ID}=\left[\frac{s_m\left(1-F\right)}{2F\left(1-{s}_m\right)}\right] $$

We estimated 95% bootstrap percentile confidence intervals for the ID estimates of each patch based on 1000 ID bootstrap values generated from the bootstrap distributions of *F* and *s*_*m*_. This alternative estimate of ID was used in one patch (patch 16; Table 2) and, more importantly, in the bootstrap analysis of ID. Use of the alternative estimate of ID when Ritland’s equilibrium estimator was <0 ensured a roughly normal distribution of ID bootstrap values. Statistical departures of estimates from 0.5 - 1 were assessed by Wilcoxon tests performed on the bootstrap distribution of ID values for each patch. Finally, we used linear regression analysis (PROC REG) to investigate the relationship between *R. ferrugineum* floral display (log), *F* estimates and ID.

## Availability of supporting data

The data sets supporting the results of this article are included within the article and its additional file.

## Additional file

Additional file 1: Table S1. Mean seed set per patch for the four pollination treatments (*IN*, *EN*, *IX* and *ISB*), pollination parameters per patch (reproductive assurance per patch, RA and self-compatibility per patch, SC) and mean fruit set per patch.
