# Supplementary material for: High lifetime inbreeding depression counteracts the reproductive assurance benefit of selfing in a mass-flowering shrub
Source: BMC Evol Biol. 2014 Nov 30;14:243. doi: 10.1186/s12862-014-0243-7 (PMC4258271; doi:10.1186/s12862-014-0243-7)
Supplement: Additional file 1: Table S1. — Mean seed set per patch for the four pollination treatments (IN, EN, IX and ISB), pollination parameters per patch (reproductive assurance per patch, RA and self-compatibility per patch, SC) and mean fruit set per patch. [file 12862_2014_243_MOESM1_ESM.docx]

**Table S1.** Mean seed set per patch for the four pollination treatments (*IN*, *EN*, *IX* and *ISB*) pollination parameters (reproductive assurance per patch, RA and self-compatibility per patch, SC) and mean fruit set per patch.

Seed sets are calculated as mean production of seeds per treatment per individual divided by mean number ovules per individual. See text for method description. *IN*: seed set from intact inflorescences allowed to undergo natural pollination; *EN*: seed set from emasculated inflorescences allowed to undergo natural pollination; *IX*: seed set from intact inflorescences outcrossed by hand; *ISB*: seed set from bagged intact inflorescences self-pollinated by hand). RA = 1 – (*ENseedset_mean* / *INseedset_mean*); SC = *ISBseedset_mean* / *IXseedset_mean*. Fruit sets are calculated as number of filled fruits divided by the total number of fruits (filled of seeds and aborted). Patches are classified from the smallest (Patch 1) to the largest patch floral display (Patch 28).

| code_patch | INseedset_mean | | ENseedset_mean | IXseedset_mean | ISBseedset_mean | RA_index | SC_index | Fruitset_mean |  |
| --- | --- | --- | --- | --- | --- | --- | --- | --- | --- |
| 1 | 0.904 | | 0.128 | 0.436 | 0.368 | 0.858 | 0.843 | 0.806 |  |
| 2 | 0.806 | | 0.270 | 0.729 | 0.574 | 0.665 | 0.788 | 1.000 |  |
| 3 | 0.677 | | 0.530 | 0.882 | 0.536 | 0.218 | 0.607 | 0.941 |  |
| 4 | 0.894 | | 0.790 | 0.999 | 0.644 | 0.116 | 0.645 | 1.000 |  |
| 5 | 0.651 | | 0.604 | 0.980 | 0.545 | 0.072 | 0.556 | 0.917 |  |
| 6 | 0.676 | | 0.513 | 0.686 | 0.365 | 0.242 | 0.532 | 0.872 |  |
| 7 | 0.631 | | 0.326 | 0.599 | 0.443 | 0.484 | 0.739 | 0.895 |  |
| 8 | 0.753 | |  | 0.640 | 0.440 |  | 0.688 | 0.974 |  |
| 9 | 0.730 | | 0.528 | 0.647 | 0.417 | 0.276 | 0.644 | 0.906 |  |
| 10 | 0.679 | | 0.779 | 0.916 | 0.797 | -0.148 | 0.870 | 0.903 |  |
| 11 | 0.698 | | 0.576 | 0.855 | 0.667 | 0.176 | 0.780 | 0.934 |  |
| 12 | 0.541 | | 0.305 | 0.395 | 0.312 | 0.437 | 0.789 | 0.878 |  |
| 13 | 0.687 | | 0.407 | 0.646 | 0.586 | 0.407 | 0.907 | 0.869 |  |
| 14 | 0.776 | | 0.489 | 0.778 | 0.502 | 0.371 | 0.645 | 0.980 |  |
| 15 | 0.628 | | 0.382 | 0.770 | 0.412 | 0.391 | 0.535 | 0.901 |  |
| 16 | 0.727 | | 0.359 | 0.704 | 0.402 | 0.506 | 0.570 | 0.951 |  |
| 17 | 0.506 | | 0.403 | 0.794 | 0.684 | 0.204 | 0.862 | 1.000 |  |
| 18 | 0.639 | | 0.451 | 0.584 | 0.466 | 0.295 | 0.798 | 0.946 |  |
| 19 | 0.687 | | 0.415 | 0.622 | 0.438 | 0.396 | 0.705 | 0.989 |  |
| 20 | 0.616 | | 0.524 | 0.755 | 0.514 | 0.151 | 0.680 | 0.977 |  |
| 21 | 0.889 | | 0.715 | 0.651 | 0.535 | 0.196 | 0.821 | 1.000 |  |
| 22 | 0.723 | | 0.492 | 0.649 | 0.383 | 0.319 | 0.590 | 0.966 |  |
| 23 | 0.767 | | 0.369 | 0.642 | 0.660 | 0.519 | 1.000 | 0.967 |  |
| 24 | 0.625 | | 0.615 | 0.617 | 0.452 | 0.017 | 0.733 | 0.990 |  |
| 25 | 0.703 | | 0.687 | 0.809 | 0.488 | 0.023 | 0.603 | 0.942 |  |
| 26 | 0.542 | | 0.614 | 0.598 | 0.397 | -0.134 | 0.663 | 0.979 |  |
| 27 | 0.562 | | 0.520 | 0.735 | 0.401 | 0.075 | 0.546 | 0.989 |  |
| 28 | 0.461 | | 0.405 | 0.679 | 0.542 | 0.121 | 0.798 | 1.000 |  |
|  | |  |  |  |  |  |  |  |  |
